# The Arabidopsis *NF-YA3* and *NF-YA8* Genes Are Functionally Redundant and Are Required in Early Embryogenesis

**DOI:** 10.1371/journal.pone.0082043

**Published:** 2013-11-26

**Authors:** Monica Fornari, Valentina Calvenzani, Simona Masiero, Chiara Tonelli, Katia Petroni

**Affiliations:** Dipartimento di Bioscienze, Università degli Studi di Milano, Milano, Italy; Umeå Plant Science Centre, Sweden

## Abstract

Nuclear factor Y (NF-Y) is a trimeric transcription factor composed of three distinct subunits called NF-YA, NF-YB and NF-YC. In *Arabidopsis thaliana*, NF-Y subunits are known to play roles in many processes, such as gametogenesis, embryogenesis, seed development, drought resistance, ABA signaling, flowering time, primary root elongation, Endoplasmic Reticulum (ER) stress response and blue light responses. Here, we report that the closely related *NF-YA3* and *NF-YA8* genes control early embryogenesis. Detailed GUS and *in situ* analyses showed that *NF-YA3* and *NF-YA8* are expressed in vegetative and reproductive tissues with the highest expression being during embryo development from the globular to the torpedo embryo stage. Plants from the *nf-ya3* and *nf-ya8* single mutants do not display any obvious phenotypic alteration, whereas *nf-ya3 nf-ya8* double mutants are embryo lethal. Morphological analyses showed that the *nf-ya3 nf-ya8* embryos fail to undergo to the heart stage and develop into abnormal globular embryos with both proembryo and suspensor characterized by a disordered cell cluster with an irregular shape, suggesting defects in embryo development. The suppression of both *NF-YA3* and *NF-YA8* gene expression by RNAi experiments resulted in defective embryos that phenocopied the *nf-ya3 nf-ya8* double mutants, whereas complementation experiments partially rescued the abnormal globular *nf-ya3 nf-ya8* embryos, confirming that *NF-YA3* and *NF-YA8* are required in early embryogenesis. Finally, the lack of GFP expression of the auxin responsive *DR5rev::GFP* marker line in double mutant embryos suggested that mutations in both *NF-YA3* and *NF-YA8* affect auxin response in early developing embryos. Our findings indicate that *NF-YA3* and *NF-YA8* are functionally redundant genes required in early embryogenesis *of Arabidopsis thaliana*.

## Introduction

In plants and animals, developmental processes are controlled by complex networks of transcription factors, often arranged in multiprotein DNA-binding complexes, whose gene regulatory activity derives from intrinsic properties and the properties of their trans-acting partners [1]–[4].

The NUCLEAR FACTOR Y (NF-Y), also called the CCAAT binding factor (CBF) and the heme-activated protein in yeast (HAP), is a heterotrimeric transcription factor that binds with high affinity and sequence specificity the highly conserved core sequence CCAAT. The CCAAT motif is one of the common *cis*-elements present in 25% of eukaryotic promoters [5], [6]. The NF-Y heterotrimer is composed of distinct subunits: NF-YA (CBF-B, HAP2), NF-YB (CBF-A, HAP3), and NF-YC (CBF-C, HAP5). NF-YB and NF-YC subunits form a tight dimer through protein structures similar to the Histon Fold Motif (HFM), a conserved protein-protein and DNA-binding interaction module [7], [8]. This dimer translocates to the nucleus, where it offers a complex surface for the association of the NF-YA subunit [9]–[12]. The resulting mature NF-Y heterotrimer is then competent to bind with high specificity and affinity the CCAAT motif in promoters [13]. NF-YA and NF-YC subunits contain large domains rich in glutamines and hydrophobic residues with transcriptional activation function [13]. The NF-YA subunit contains a conserved domain (HAP2-homology domain) at the C-terminal of the protein, which is divided into two sub-domains: a Subunit Association Domain (the N-terminal part required for NF-YB-NF-YC association) and a DNA binding domain (the C-terminal part required for DNA binding), separated by a linker region [13], [14]. In animals and yeast, each subunit of NF-Y is encoded by a single gene, whereas in the Arabidopsis genome 10 NF-YAs, 10 NF-YBs and 9 NF-YCs are annotated [1], [14]–[16]. Recently, a reclassification of the plant *NF-Y* genes, based on Arabidopsis NF-Y nomenclature, aimed to avoid confusion with acronyms and related, but functionally distinct, histone fold domain (HFD) protein was proposed [16]. Furthermore, the conserved heterodimerization capacity of At-NF-Y histone-like subunits and the different affinities of At-NF-YAs for the CAAT sequence was determined [17].

Genetic and physiological studies have shown that NF-Ys in plants have several different functions. It has been demonstrated that the Arabidopsis *NF-YB* and *NF-YC* genes are involved in gametogenesis, embryogenesis, seed development, drought resistance, ABA signaling, flowering time and primary root elongation [18]-[27]. *NF-YA* genes are involved in drought resistance [28], in Endoplasmic Reticulum (ER) stress response and in blue light and ABA responses with *NF-YB* and *NF-YC* [21], [29].

Embryogenesis in higher plants is a developmental process divided into three distinct phases: morphogenesis, maturation phase and late embryogenesis [30], [31]. During the morphogenesis phase, the basic body plan of the plant is established [32], [33]. Following fertilization, the zygotic division generates a smaller apical and a larger basal cell. The apical cell divides vertically and generates a proembryo; the basal cell of the zygote divides transversely to produce the hypophysis and the extraembryonic suspensor [34]. Each cell derived from the apical cell divides periclinally to produce a 16-cell embryo (early globular stage). Then, the cells continue to undergo division producing 32-cell (midglobular stage) and 64-cell stage embryo (late globular stage); at this time cell division in the protoderm increases producing the cotyledon primordia and leads to the heart stage embryo. Then, after cell divisions and cells elongation, the embryo assumes an elongated shape, typical of the torpedo stage [35]. The embryo now enters into the maturation phase: the torpedo stage embryo elongates into a “walking stick” embryo, then the cotyledons grow and curve over forming the “upturned-U” embryo [35]. Finally, the late embryogenesis phase allows the fully developed embryo to enter a desiccated and metabolically quiescent state.

Developmental pathways are involved in the early events of embryo development and they are linked to apical-basal embryo axis establishment after zygote division. One involves the transcription factors WUSCHEL RELATED HOMEOBOX (WOX) WOX8, WOX9 and WOX2; mutations in the *WOX* genes determine defects in the zygote stage [36]-[39]. Another developmental pathway is auxin dependent. Auxin is a signalling hormone implicated in the establishment of the embryonic body axis [40] which undergoes polar transport mediated by asymmetric localization of specific cellular efflux carrier proteins, the PINFORMED (PIN) protein family [41], [42]. Immediately after the zygote division, auxin is localized in the apical cell and then in the developing proembryo. At the 32-cell stage it undergoes a basal shift to the hypophysis and finally, at later stages of embryogenesis, is also concentrated in the tips of cotyledons and in the provascular strands [43]. It is known that interfering with auxin transport causes defects in the determination of the apical-basal poles during embryo development [43]–[45]. Axialization of the embryo requires also *AUXIN RESPONSE FACTOR* (*ARF*) genes: *MONOPTEROS* (*MP*)*/AUXIN RESPONSE FACTOR 5* (*ARF5*) and its *AUXIN/INDOLE-3-ACETIC ACID* (*AUX/IAA*) inhibitor *BODENLOS* (*BDL*)/*IAA12*. It is known that *mp* and *bdl* embryos show defects in the embryonic axis [46], [47].

In this paper, we present the functional characterization of *At-NF-YA3* and *At-NF-YA8*, two members of the Arabidopsis *NF-YA* family, very closely related in the phylogenetic tree [1], [15]. We show that *NF-YA3* and *NF-YA8* are expressed in vegetative and reproductive tissues, in particular during embryo development from globular to torpedo stage. Plants from single *nf-ya3* and *nf-ya8* mutants are similar to wild-type plants, whereas *nf-ya3 nf-ya8* double mutants are embryo lethal. Nomarski microscopy analysis show that *nf-ya3 nf-ya8* fail to undergo to the heart stage and develop into abnormal globular embryos with both proembryo and suspensor characterized by a disordered cell cluster with an irregular shape. Our data indicate that *NF-YA3* and *NF-YA8* are required in early embryogenesis.

## Materials and Methods

### Plant materials and growth conditions

Wild-type *Arabidopsis thaliana* seeds (Col-O) and *DR5rev::GFP* [48] seeds were obtained from the Nottingham Arabidopsis Stock Centre, UK (NASC). The *nf-ya3* (SAIL_138_E04 line) and *nf-ya8* (SAIL_759_B07 line) T-DNA insertion mutants were found in the SAIL collection (http://signal.salk.edu/cgi-bin/tdnaexpress). Plants were grown in a 22°C±2°C temperature growth room under long-day conditions (14 h light/10 h dark) at a fluence rate of 100 µE m^−2^s^−1^.

### In situ hybridization

Flowers and siliques of wild-type Arabidopsis plants were collected at various developmental stages and were fixed and embedded in Paraplast Plus embedding media as described by Cañas et al. (1994). Digoxygenin-labeled antisense and sense RNA probes were generated by in vitro transcription according to the instructions of the DIG RNA Labeling Kit (SP6/T7; Roche, http://www.roche.com). cDNA used for probe transcription was synthetized by PCR with the following primers: a forward primer A3NoT7a (5′-GTTAGCCAGAGAGCCTTATTC-3′) and a reverse primer containing a T7 promoter adapter A3+T7a) (5′-TAATACGACTCACTATAGGGGTAATCGATGAAGCGAAGAAG-3′) for *NF-YA3*; a forward primer A8NoT7-A2 (5′-GCTAATTGTTGCCTCTGAG-3′) and a reverse primer containing a T7 promoter adapter A8+T7-a (5′-TAATACGACTCACTATAGGGGAATCCTGGTCCTGGAAAC-3′) for *NF-YA8*. cDNA used for sense probe transcription was synthetized by PCR with the following primers: a forward primer containing a T7 promoter adapter A3T7S (5′-TAATACGACTCACTATAGGGGTTAGCCAGAGAGCCTTATTC-3′) and a reverse primer A3noT7S (5′-GTAATCGATGAAGCGAAGAAG-3′) for *NF-YA3*; a forward primer containing a T7 promoter adapter A8T7-S2 (5′-TAATACGACTCACTATAGGGGCTAATTGTTGCCTCTGAG-3′) and a reverse primer A8noT7S (5′-GAATCCTGGTCCTGGAAAC-3′) for *NF-YA8*. Hybridization and immunological detection were performed as described previously [49]. The sections were mounted with a coverslip and subsequently observed using a Zeiss Axiophot D1 microscope equipped with differential interface contrast (DIC) optics. Images were captured on an Axiocam MRc5 camera (Zeiss) using the AXIOVISION program (version 5.0).

### Constructs and generation of transgenic lines

The *pNF-YA3::GUS* fusion was constructed from a 1995-bp genomic region upstream of the *NF-YA3* start codon including the entire upstream intergenic region amplified by PCR with a forward primer containing a *Sal*I restriction site at the 5′ end (5′-GTCGACACGGCTTTGTAGAG-3′) and a reverse primer containing a *Xba*I site at the 3′ end (5′-TCTAGAACGTCTTCCTTAACA-3′). The *pNF-YA8::GUS* fusion was constructed from a 1000-bp genomic region upstream of the *NF-YA8* start codon including the upstream intergenic region and 49 bp of the 3′ UTR of At1g17600 gene amplified by PCR with a forward primer containing a *Sal*I restriction site at the 5′ end (5′-GTCGACGACTTAAACTGTGTGC-3′) and a reverse primer containing a *Xba*I site at the 3′end (5′-TCTAGACAACAATTAGCTCTCT-3′). The PCR fragments were cloned in the pCR4-TOPO (Invitrogen), excised with *Sal*I - *Xba*I and subcloned between the *Sal*I - *Xba*I sites preceding the *GUS* gene in the binary vector pGPTV-Kan [50].

The constructs for *35S::NF-YA3* and *35S::NF-YA8* lines were obtained using the Gateway cloning system. *NF-YA3* and *NF-YA8* cDNAs were amplified by PCR using the following primers: A3TH5 (5′-AATTCCAGCTGACCACCATGATGCATCAGATGTTGA-3′) and A3TH3 (5′-GATCCCCGGGAATTGCCATGTCAGATATGGACAGAG-3′) for *NF-YA3* and A8TH5 (5′-AATTCCAGCTGACCACCATGGATAAGAAAGTTTCAT-3′) and A8TH3 (5′-GATCCCCGGGAATTGCCATGTCAGATATGGACAGAG-3′) for *NF-YA8*. The PCR products were cloned in the binary vector in the pGD625 vector under the Cauliflower Mosaic Virus 35S promoter (35S) according to manufacturer instructions (Gateway, Invitrogen).

For the RNA interference (RNAi) construct, a 200-bp specific fragment for *NF-YA8* that shared 80% of identity with *NF-YA3* gene was amplified by PCR using the forward primer 8-3 RNAi F (5′-GCGTTAATCTCTTTGGACAC-3′) in combination with the reverse primer 8-3 RNAi R (5′-AGTAGCAGCCAAGGATGACT -3′). The fragment was placed in both orientations into the pFRH destination vector (derived from pFGC5941; NCBI accession number AY310901) under the control of the *35S* promoter using the Gateway technology (Invitrogen). All the constructs were introduced into *A. tumefaciens* GV3101 [51].

The *pNF-YA3::GUS* and *pNF-YA8::GUS* constructs were then transferred to wild-type *Col* and the *35S::NF-YA3* and *35S::NF-YA8* constructs to *nf-ya3*/*nf-ya3 NF-YA8/nf-ya8* plants by the floral dip method [52]. Homozygous T_3_ independent lines with a single T-DNA insertion were selected by segregation analysis on MS agar plates supplemented with 1% (w/v) sucrose containing 50 µg ml^−1^ kanamycin.

The RNAi construct was transferred to *nf-ya3* and *nf-ya8* single mutants and then T_1_ independent *RNAi::A3/A8* lines were selected on MS agar plates supplemented with 1% (w/v) sucrose containing 20 µg ml^−1^ hygromycin.

To analyse auxin response in *nf-ya3 nf-ya8* embryos, plants *nf-ya3*/*nf-ya3 NF-YA8/nf-ya8* used as females were crossed with the *DR5rev::GFP* marker line used as male. From the F_1_ progeny, plants *nf-ya3*/*nf-ya3 NF-YA8/nf-ya8* were selected by PCR genotyping and self-fertilized. The resulting F_2_ plants were analysed by visual inspection of GFP signal in roots using an Olympus SZX12 stereomicroscope, in order to select those carrying the *DR5rev::GFP* construct, and then analysed by PCR genotyping to select those with *nf-ya3*/*nf-ya3 NF-YA8/nf-ya8* genotype. Selected F_2_ plants were then self-fertilized and the resulting F_3_ plants were analysed by visual inspection of GFP signal in root, in order to identify plants homozygous for the *DR5rev::GFP* construct.

### GUS staining assay

For detection of GUS activity, vegetative tissues and flowers at different stages were incubated for 16 hr at 37°C in the GUS staining buffer (0.5 mg/ml X-glucuronic acid, 0.1% Triton X-100, and 0.5 mM ferrocyanidine in 100 mM phosphate buffer, pH 7). Longitudinally dissected siliques were fixed for 2 hr at −20°C in 90% acetone and subsequently immersed in GUS staining buffer for 16 hr at 37°C. Vegetative tissues were cleared in 70% ethanol, flowers and siliques were cleared in a solution of chloral hydrate:glycerol:water (8∶1∶2, w∶v∶v). GUS staining in ovule, pollen and embryos at different stages was observed under Nomarski optics on a Zeiss Axio Imager.D1 microscope with a video camera Axiocam MRc5. GUS staining in other tissues was examined under a stereomicroscope LEICA® MZ6 and the GUS images were taken with LEICA® DC 500 camera.

### Genotyping of mutants and transgenic plants by PCR

Genomic DNA was extracted from plants as previously described [53]. Wild-type alleles were amplified using the following primers: atACF3 (5′-CTCTGCAATCGTGTTTTGGTT-3′) and atACR1 (5′-GTTAGGCCAACAGGTAAACAG-3′) for the *NF-YA3* allele and NF-YA8F2 (5′-CTTTGACAGACACATGTATCATCAA-3′) and NF-YA8R2 (5′-TGGCTGCTACTTCGCTTATTAGC-3′) for the *NF-YA8* allele. The *nf-ya3* and *nf-ya8* mutant alleles were amplified using the following primers: the T-DNA left border-specific primer LB3 (5′-TAGCATCTGAATTTCATAACCAATCTCGATACAC-3′) together with the *NF-YA3*-specific primers atACR1 for the *nf-ya3* allele or with the *NF-YA8*-specific primer NF-YA8R2 for the *nf-ya8* allele.

The presence of the complementation constructs was verified by PCR with a forward primer 35S (5′-TCTAGACAAGACCCTTCCTC -3′) in combination with the reverse primer atACR1 for the *35S::NF-YA3* construct or with the reverse primer NF-YA8R2 for the *35S::NF-YA8* construct.

### Whole mount preparation and confocal laser scanning

Whole mount embryos were prepared by clearing siliques at different stages of *nf-ya3*/*nf-ya3 NF-YA8/nf-ya8* plants in chloral hydrate:glycerol:water solution (8∶1∶2, w∶v∶v). Siliques were dissected under the stereomicroscope and observed under Nomarski optics. Confocal laser scanning microscopy was performed on embryos collected from *nf-ya3*/*nf-ya3 NF-YA8/nf-ya8* plants homozygous for the *DR5rev::GFP* construct using Leica TCS SP5 AOBS and DMI 6000 CS microscope.

### Semi-quantitative RT-PCR analysis

Total RNA was isolated [54] from developing siliques of *nf-ya3* and *nf-ya8* plants and of *35S::NF-YA3* and *35S::NF-YA8* overexpression lines as previously described [14]. Approximately 5 µg were reverse-transcribed using RT Superscript II (Invitrogen) and an oligo dT, as previously described [55]. After first strand cDNA synthesis, samples were diluted 50 times and used as templates for semi-quantitative RT-PCR, with *NF-YA3*-specific primers atACF2 (5′-ATCTTATACTGAAGTTGCTAGTAG-3′) and atACR2 (5′-TTCTGCAGCATATGAGCTTCGA-3′) and *NF-YA8*-specific primers NF-YA8F2 and NF-YA8R2. *ACTIN2* specific primers, ACT2-F (5′-TGCTTCTCCATTTGTTTGTTTC-3′) and ACT2-R (5′-GGCATCAATTCGATCACTCA-3′) were used as a control of cDNA concentration. The amplifications were carried out within linear ranges (25 cycles). The PCR products were transferred onto Hybond N+ nylon membranes (Amersham) and hybridized with gene-specific probes labelled using the DIG-High Prime kit (Roche).

### Quantitative real-time RT-PCR analysis

Total RNA was isolated from siliques of the *RNAi::A3/A8* transgenic plants and the cDNA was synthesized as above. Quantitative real-time RT-PCR of *NF-YA3* and *NF-YA8* was performed using SYBRGreen with the Cfx96TM BioRad® Real Time system in a final volume of 20 µl containing 5 µl of 50-fold diluted cDNA, 0.25–0.4 µM of each primer and 10 µl of 2X SOS Fast™ EVA-Green® Supermix (BioRad Laboratories, Hercules, CA, USA).

Analysis of *NF-YA3* and *NF-YA8* expression in *RNAi::A3/A8* lines was performed using *NF-YA3*-specific primers NFYA3_RT-F and NFYA3_RT-R and *NF-YA8*-specific primers NFYA8_RT-F (5′-CACACACCATTTCTCTGTCCA-3′) and NFYA8_RT-R (5′- TTGAAGCTTGAAGGTTTTTGG -3′). *ACTIN2* and *RCE1* were used as the reference genes. Specific primers for *ACTIN2* gene are ACT2 F and ACT2 R (see above), for *RCE1*we used RT 147 (5′-CTGTTCACGGAACCCAATTC-3′) and RT 148 (5′-GGAAAAAGGTCTGACCGACA-3′) [56].

Relative quantification was analysed using iCycler™ iQ Optical System Software version 3.0a (BioRad Laboratories). The protocol used was as follows: 95°C for 2 min, 55 cycles of 95°C for 15 s, 60°C for 30 s. A melt curve analysis was performed following every run to ensure a single amplified product for every reaction (from 55°C to 95°C, increment 0.5°C for 10 sec).

Relative quantification of the target RNA expression level was performed using the comparative Ct method (User Bulletin 2, ABI PRISM7700 Sequence Detection System, December 1997; Perkin Elmer Applied Biosystems) in which the differences in the Ct (threshold cycle) for the target RNA and endogenous control RNA, called ΔCt, were calculated to normalise for the differences in the total amount of cDNA present in each reaction and the efficiency of the reverse transcription. Finally, the target RNA expression level was obtained from the equation 2^−ΔΔCt^ and expressed relative to a calibrator (wild-type siliques). Standard errors of ΔΔCt values were obtained from measurements performed in triplicate.

## Results

### The duplicated genes NF-YA3 and NF-YA8 have similar expression patterns

**Figure 1:**
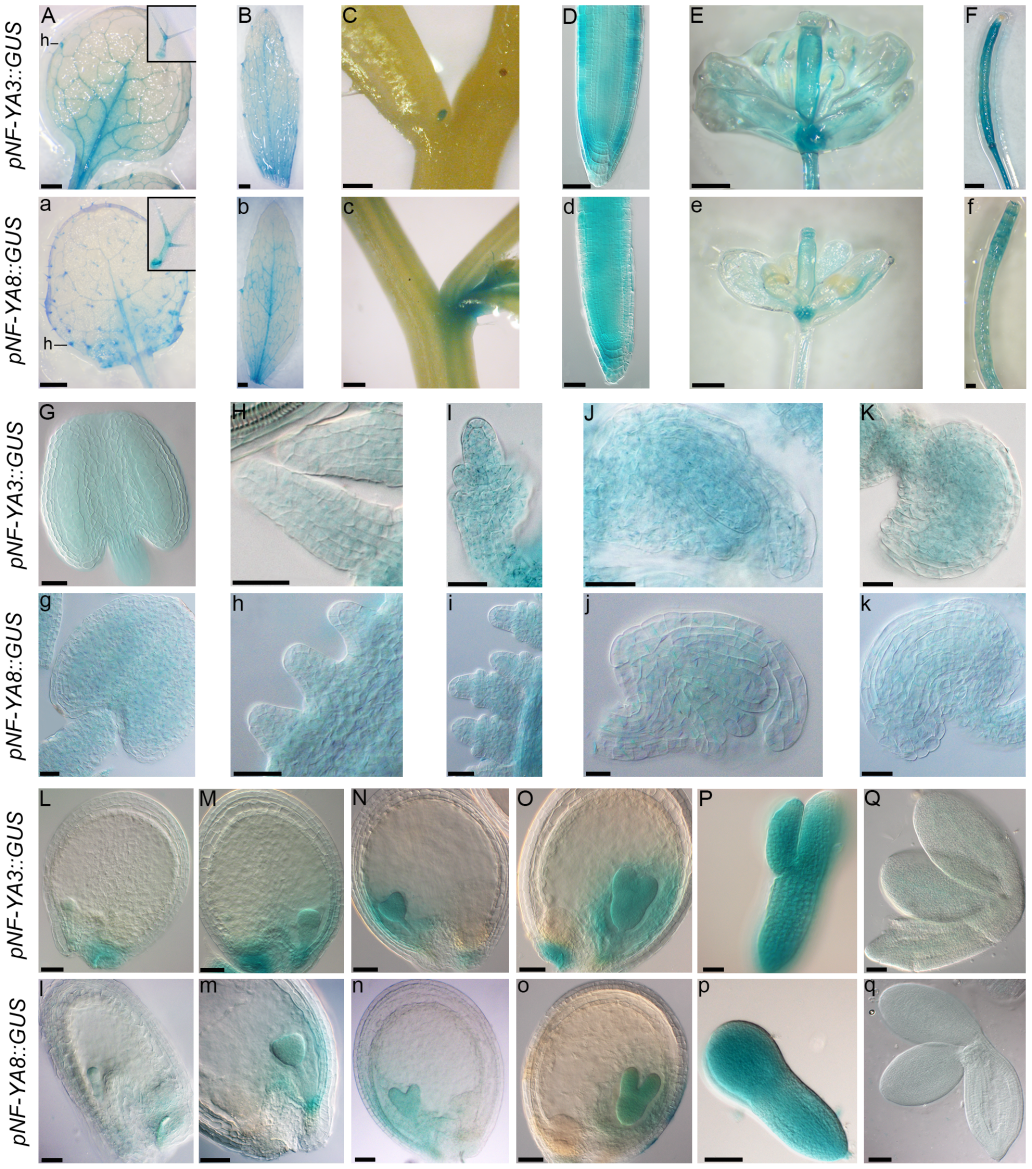
GUS staining in *pNF-YA3::GUS* and *pNF-YA8::GUS* plants. In capital letters GUS staining in *pNF-YA3::GUS* plants; in small letters GUS staining in *pNF-YA8::GUS* plants. GUS staining in vegetative and reproductive tissues: in rosette leaves (A and a), in cauline leaves (B and b), in axils of stem branches (C and c), in roots (D and d), in flowers (E and e) and in siliques (F and f). (G and g) GUS staining in anthers at 8 floral stage. (H–K and h–k) GUS staining in the different stages of ovule development. (H and h) ovule primordia of flower at stage 9–10, (I and i) developing ovules of flower at stage 11, (J and j) ovule of flower at stage 12 and (K and k) mature ovule of flower at stage 13. (L–Q and l–q) GUS staining during seed development. (L and l) Embryo at 2–4 cell stage, (M and m) globular, (N and n) mid-heart, (O and o) late-heart, (P and p) torpedo and (Q and q) mature embryo. Stages of flower development are according to Bowman, 1994. Insets in A, a show higher magnification of trichomes. Abbreviations: h, hydathodes. Bars in (A) to (C), (a) to (c) and (E), (F), (e), (f) = 250 µm; bars in (G) to (K) and (g) to (k) = 20 µm; bars in (D) and (d), (L) to (Q) and (l) to (q) = 50 µm.

Previous expression analyses showed that the *NF-YA3* and *NF-YA8* genes are both expressed in vegetative and reproductive tissues during flower and seed development [14], [15]. Furthermore, *NF-YA3* and *NF-YA8* share a high sequence similarity. Overall, they are 62% identical at the amino acid level, with 93% identity shared between their conserved domain (HAP2-homology domain) at the C-terminal of the protein. Analysis of their chromosomal locations revealed that they lie within a relatively recent segmental duplicated region of the Arabidopsis genome [57], which is proposed to have occurred prior to the Arabidopsis-Brassica split (12–20 Mya) [58]. In order to characterize the *NF-YA3* and *NF-YA8* genes in Arabidopsis, we first analysed in detail their expression pattern in different organs and developmental stages. To this purpose, we obtained transgenic plants carrying a 1995 bp genomic region upstream of *NF-YA3* start codon fused to the *GUS* reporter gene (*pNF-YA3::GUS*) and plants carrying a 1000 bp genomic region upstream of *NF-YA8* start codon fused to the *GUS* reporter gene (*pNF-YA8::GUS*). In total, 7 independent transgenic lines for the *pNF-YA3::GUS* construct and 6 for the *pNF-YA8::GUS* construct were analysed. For both constructs, five transgenics showed similar GUS expression patterns in vegetative and reproductive tissues (Fig. 1). In particular, a similar GUS staining was detected in rosette leaves, which showed high signal in midrib, hydathodes and trichomes (Fig. 1 A,a). GUS staining was also observed in cauline leaves (Fig. 1 B,b), in roots (Fig. 1 D,d), in all flower organs (Fig. 1 E,e) and in siliques (Fig. 1 F,f). In axils of stem branches, *NF-YA8* was more expressed than *NF-YA3* (Fig. 1 C,c). Detailed analyses of GUS expression in reproductive organs revealed similar expression of *NF-YA3* and *NF-YA8* in anthers and in pollen grains at floral stage 8 according to Bowman, 1994 (Fig. 1 G,g), whereas in subsequent stages the signal was in filaments of anthers, but not in mature pollen grains (data not shown). GUS staining was detected in ovules at all developmental stages (Fig. 1 H–K and h–k). After fertilization, there was a low GUS signal level for both constructs in the embryo at the 2–4 cell stage (Fig. 1 L,l), then the signal increased and was high from the globular to the torpedo stages (Fig. 1 M–P and m–p), after which GUS staining decreased (Fig. 1 Q,q).

**Figure 2:**
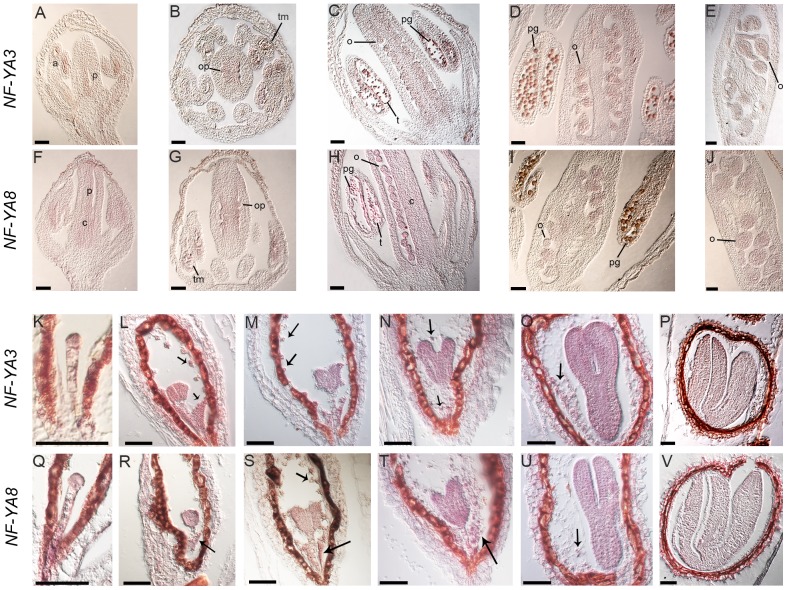
In situ analyses of *NF-YA3* and *NF-YA8* expression in developing flower and seed. Longitudinal sections of developing flowers, showing *NF-YA3* (A-E) and *NF-YA8* (F–J) expression in flower at stage 8 (A,F), in flower at stage 9 (B,G), stage 11 (C,H), 12 (D,I) and stage 13 (E,J). Stages of flower development are according to Bowman, 1994. Longitudinal sections of developing seeds, showing *NF-YA3* (K–P) and *NF-YA8* (Q–V) expression in different stages of embryo development: (K,Q) embryo at early globular stage, (L,R) globular, (M,S) mid-heart, (N,T) late-heart, (O,U) torpedo and (P,V) cotyledon stage. Arrows indicate endosperm cells. Abbreviations: p, placenta; c, carpel; op, ovule primordia; o, ovule; a, anther; tm, tetrad of microspores; pg, pollen grains; t, tapetum. Bars = 50 µm.

To further investigate the temporal and spatial expression profile of *NF-YA3* and *NF-YA8* during flower and seed development, we performed *in situ* hybridization analyses on developing flowers and siliques (Fig. 2). Similar weak hybridization signals were observed during ovule and pollen development. *NF-YA3* and *NF-YA8* were both expressed in the placenta (Fig.2 A,F), in the ovule primordia and in the septum (Fig. 2 B,G) and in subsequent stages of ovule development (Fig. 2 C–E and H–J). In addition, *NF-YA8* was also expressed in carpels (Fig. 2 F,H). Both genes were expressed in anthers at floral stage 8 (Bowman,1994) (Fig. 2 A,F), in tetrads of microspores (Fig. 2 B,G), in developing pollen grains and in tapetum (Fig. 2 C,H). However, no expression was observed in mature pollen grains (Fig. 2 D,I). The *NF-YA3* and *NF-YA8* transcripts were also detected during embryo development. *In situ* analysis revealed a low signal for both genes in the embryo at the early globular stage (Fig. 2 K,Q). The expression level increased in the globular stage (Fig. 2 L,R) and was high until the torpedo stage (Fig. 2 M–O and S–U); in particular, in these stages, *NF-YA3* and *NF-YA8* were expressed both in embryo and in endosperm cells. In the cotyledon stage (Fig. 2 P,V), the expression decreased and was restricted to the embryo. Similar hybridization experiments performed with *NF-YA3* and *NF-YA8* sense probes on developing flowers and seeds gave no signal (Figure S1).

Overall, the *in situ* analyses essentially confirmed the results obtained by the GUS expression analysis and showed that *NF-YA3* and *NF-YA8* have an overlapping expression profile, with high levels of transcript in the embryo from globular to torpedo stages. Furthermore, these expression analyses make them strong candidates for being functionally redundant during embryo development.

### Isolation and characterization of T-DNA insertional mutants in the NF-YA3 and NF-YA8 genes

To determine the function of the Arabidopsis *NF-YA3* and *NF-YA8* genes, we undertook a reverse genetic approach and isolated T-DNA mutant alleles for both *NF-YA3* and *NF-YA8*.

**Figure 3:**
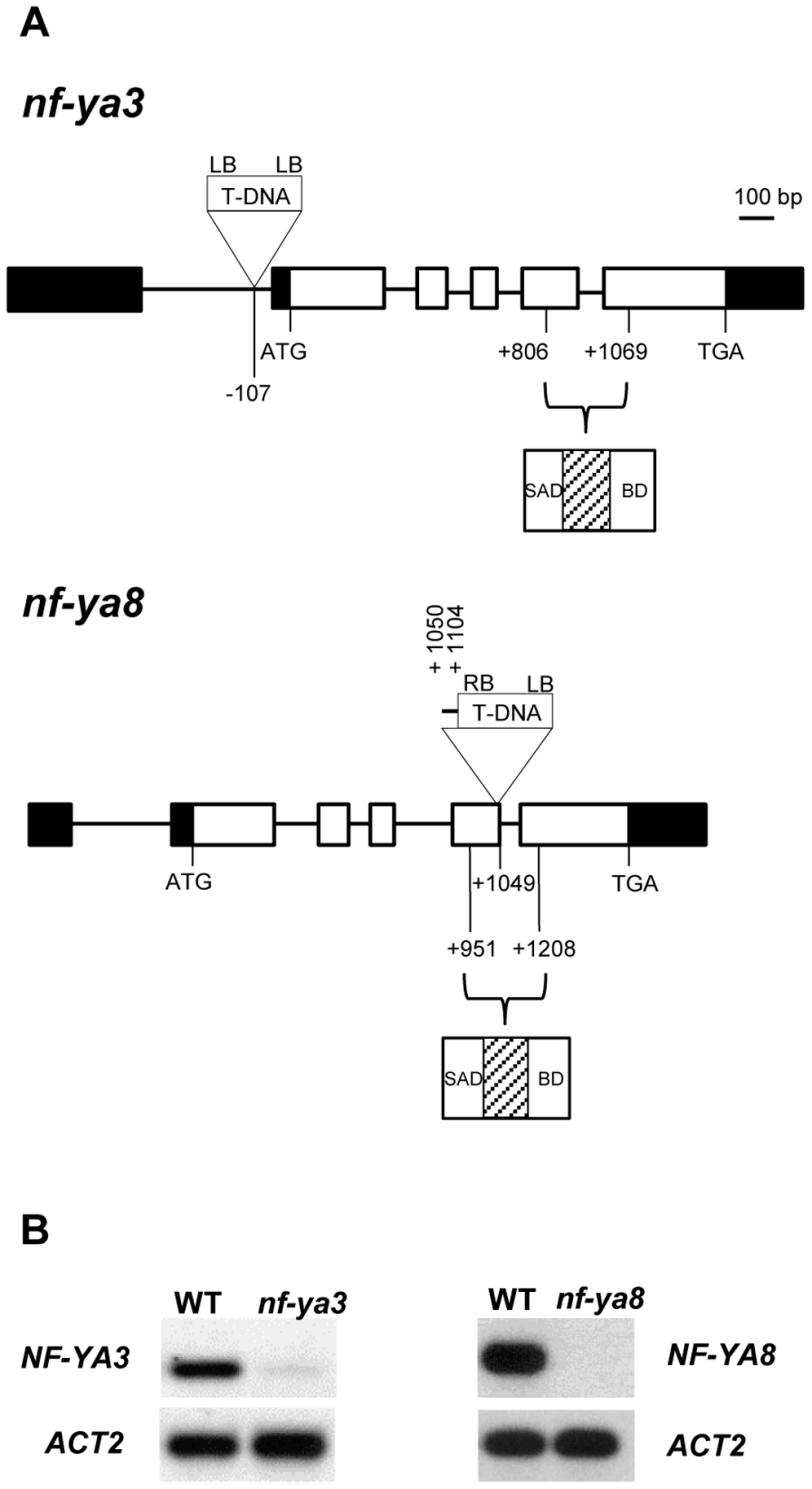
Structure of the T-DNA insertional mutants and semi-quantitative RT-PCR analyses. A) Schematic representation of the *NF-YA3* and *NF-YA8* genes. Position of the T-DNA insertion sites for *nf-ya3* and *nf-ya8* mutant alleles are indicated. Exons are boxed and lines between boxes represent introns. White boxes correspond to coding sequence exons, black boxes correspond to 5′ UTR and 3′UTR. LB, left border; RB, right border. As determined by sequencing of the T-DNA/gene junctions, the duplicated genomic region of 54 nucleotides in the *nf-ya8* mutant allele is shown. The highly conserved domain, composed by Subunit Association Domain (SAD) and DNA binding domain (BD) and the linker region (lined box), is indicated. B) RT-PCR of *NF-YA3* and *NF-YA8* transcript in *nf-ya3* and *nf-ya8* mutants. Total RNA was extracted from developing siliques of wild-type and mutant plants and analyzed by RT-PCR. All cDNA samples were standardized using a set of primers specific for the *ACTIN 2* (*ACT2*) gene.

We identified one T-DNA insertion line in *NF-YA3* in the SAIL collection. Sequencing of PCR products obtained from the T-DNA/gene junctions revealed that *nf-ya3* carries a T-DNA element located 107 bp upstream of the start codon (SAIL_138_E04) corresponding to the intron of the 5′ untranslated region (5′ UTR) (Fig. 3 A). Both the T-DNA/gene junctions could be amplified with the left border primer, implying that two or more T-DNAs had inserted into this locus as tandem inverted repeats. RT-PCR analysis showed a strong reduction of *NF-YA3* transcript levels in *nf-ya3* developing siliques compared to wild-type (Fig. 3 B). However, there were no obvious morphological alterations in *nf-ya3* mutants and genotypic analysis of 247 progeny plants from *NF-YA3*/*nf-ya3* heterozygotes revealed that the genotypic ratios were not statistically different from the 1∶2∶1 expected ratios (69∶110∶68, χ^2^ = 2.96, 0.25>P value>0.1).

One insertional mutant in *NF-YA8* was found in the SAIL collection (SAIL_759_B07). Genomic PCR and sequencing of T-DNA/gene junctions revealed that a T-DNA had inserted 1049 bp downstream of the *NF-YA8* start codon in the linker region of the conserved domain between the two sub-domains (Subunit Association Domain and DNA binding domain).

Furthermore, sequencing of *NF-YA8* T-DNA flanking regions also showed a duplicated genomic region of 54 bp close to the RB border (corresponding to the sequence comprised between position +1050 bp and +1104 bp from the start codon) (Fig. 3 A). RT-PCR analysis showed that the *NF-YA8* transcript was absent in *nf-ya8* homozygous developing siliques (Fig. 3 B). However, the knock-out mutation of *NF-YA8* did not cause any obvious morphological defect and the segregation analysis of 158 progeny plants from *NF-YA8*/*nf-ya8* heterozygotes showed a normal genotypic 1∶2∶1 ratio (51∶69∶38, χ^2^ = 4.67, P value ≈0.1).

### Double mutants nf-ya3 nf-ya8 are embryo-defective

Since characterization of *nf-ya3* and *nf-ya8* single mutants did not show any phenotypic defect, in order to verify whether *NF-YA3* and *NF-YA8* were redundant genes, we aimed to generate a *nf-ya3 nf-ya8* double mutant. Therefore, a cross was made between *nf-ya3* and *nf-ya8* homozygous plants. The resulting double heterozygous F1 plants were self-fertilized and the F2 progeny plants analysed in order to select the double mutants expected at 1/16 frequency. PCR genotyping of 81 F2 progeny plants, however, did not identify any *nf-ya3 nf-ya8* double mutants. To increase the expected frequency of recovering plants with the double mutant genotype, F3 progeny from plants that were homozygous for the *nf-ya3* mutation and heterozygous for the *nf-ya8* mutation were analyzed. One quarter of the progeny from these plants was expected to be double mutants. However, genotyping of 344 F3 individuals derived from a *nf-ya3*/*nf-ya3 NF-YA8/nf-ya8* plant failed to identify any double mutant (Table 1). In addition, the observed ratio between the other genotypic classes (217∶127, Table 1) was consistent with a 2∶1 ratio (χ^2^ = 1.986, 0.2>P>0.1). These results suggest that double mutants are embryo lethal and the presence of only one copy of *NF-YA3* or *NF-YA8* is sufficient to result in normal embryo development.

**Table 1:** Segregation analysis of F3 progeny from *nf-ya3/nf-ya3 NF-YA8/nf-ya8* plants.

Progeny genotype	Observed/expected	Observed/expected (%)
*nf-ya3/nf-ya3 NF-YA8/NF-YA8*	127/86.25	36.92/25
*nf-ya3/nf-ya3 NF-YA8/nf-ya8*	217/172.5	63.08/50
*nf-ya3/nf-ya3 nf-ya8/nf-ya8*	0/86.25	0/25
Total	344/344	100/100
P value^a^	0.2>P>0.1	

a Chi-square test for a 2∶1 segregation hypothesis.

**Figure 4:**
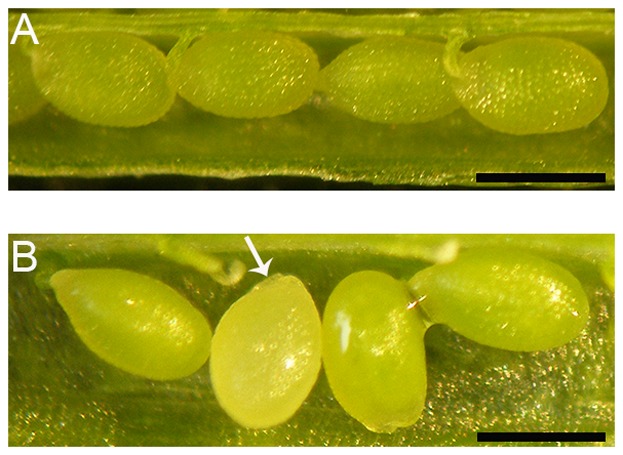
Phenotypic analysis of *nf-ya3/nf-ya3 NF-YA8/nf-ya8* siliques. A) Siliques of wild-type plants with normal seeds. B) Siliques of *nf-ya3/nf-ya3 NF-YA8/nf-ya8* mutant plants with arrested seeds (arrow). Bar = 500 µm.

Siliques of 17 plants *nf-ya3/nf-ya3 NF-YA8/nf-ya8* and siliques of 10 plants *nf-ya3/nf-ya3 NF-YA8/NF-YA8*, among F3 progeny, were analysed phenotypically. For each plant, 10 siliques were collected and observed. Siliques obtained from the *nf-ya3/nf-ya3 NF-YA8/nf-ya8* plants contained white-colored arrested seeds (Fig. 4). We found 546 arrested seeds out of 2272 seeds analysed, corresponding to a percentage of 24.03%, that is not statistically different from the expected 25% corresponding to the *nf-ya3/nf-ya3 nf-ya8/nf-ya8* genotypic class (χ^2^ = 1.136, 0.5>P>0.2) (Table 2). On the contrary, in siliques obtained from *nf-ya3/nf-ya3 NF-YA8/NF-YA8* plants, we found a percentage of arrested seeds (0.095%, n = 3163), which is comparable to those observed in wild-type siliques (0.099%, n = 1008) (Fig. 4). Therefore, these data indicate that there is a cosegregation between the genotypic class *nf-ya3/nf-ya3 nf-ya8/nf-ya8* and the phenotype of arrested seeds.

**Table 2:** Percentage of normal and arrested seeds of *nf-ya3/nf-ya3 NF-YA8/nf-ya8* mutant plants.

Seeds	Observed/expected	Observed/expected (%)
normal	1726/1704	75.97/75
arrested	546/568	24.03/25
Total	2272/2272	100/100
P value^a^	0.5>P>0.2	

a Chi-square test for a 3:1 phenotypic segregation hypothesis.

### NF-YA3 and NF-YA8 control the suspensor and embryo proper development

**Figure 5:**
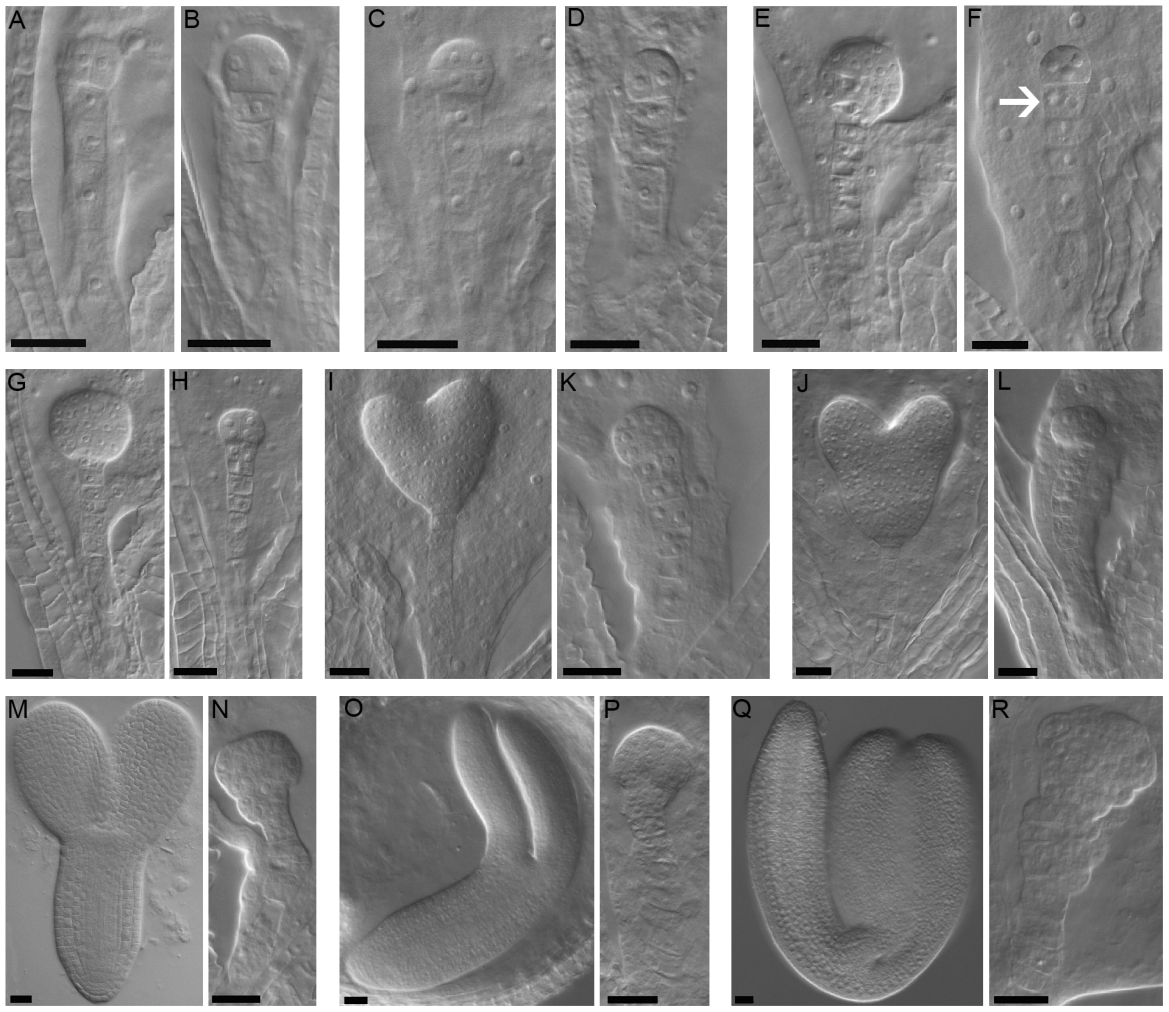
Defects in embryogenesis in *nf-ya3 nf-ya8* double mutants. Nomarski images of *nf-ya3/nf-ya3 NF-YA8/nf-ya8* siliques collected at different stages. Cleared wild-type or *nf-ya3/nf-ya3 NF-YA8/nf-ya8* seeds (A,C,E,G,I,K,M,O,Q) and *nf-ya3/nf-ya3 nf-ya8/nf-ya8* mutant seeds (B,D,F,H,J,L,N,P,R). (A,B) 4-cell stage, (C,D) 8-cell stage, (E,F) 32-cell stage, (G,H) globular stage, (I–J) mid-heart stage, (K–L) late-heart stage, (M,N) torpedo stage, (O,P) late-torpedo stage, (Q,R) cotyledon stage. Arrow indicates cells that have undergone abnormal division. Bars = 10 µm.

To determine the developmental origin of the embryo lethality, we analyzed whole-mount preparations of developing siliques after self-fertilization of *nf-ya3/nf-ya3 NF-YA8/nf-ya8* mutant plants. In particular, our aim was to identify the precise stage of embryo development which is impaired in *nf-ya3 nf-ya8* double mutants. Morphological characterization of developing siliques confirmed the presence of abnormal embryos (Fig. 5). Until 4-cell stage, the mutant embryos were normal (Fig. 5 A,B), whereas phenotypic deviations from the wild-type were first identified in embryos at the 8-cell stage, when the double mutant embryos presented a delay in cell divisions (Fig. 5 C,D). Embryos at 32-cell stage showed a hypophysis cell (uppermost region of the suspensor) with a longitudinal, instead of a transversal, division plan as found in wild-type and an abnormal number of proembryo cells caused by defects in cell division plans (Fig. 5 E,F). Afterwards, double mutant failed to undergo to the heart stage; in particular, the embryos proceeded extremely slowly and abnormally through the other stages of development (Fig. 5 G–N), and only very late in development they became an abnormal globular mass with proembryos and suspensors characterized by a disordered cell cluster with an irregular shape (Fig. 5 P,R). These data suggest that mutations in *NF-YA3* and *NF-YA8* compromise embryo development pattern and, in particular, they are required for both suspensor and embryo proper development and embryo survival.

### RNAi suppression of NF-YA3 and NF-YA8 causes embryonic defects

**Figure 6:**
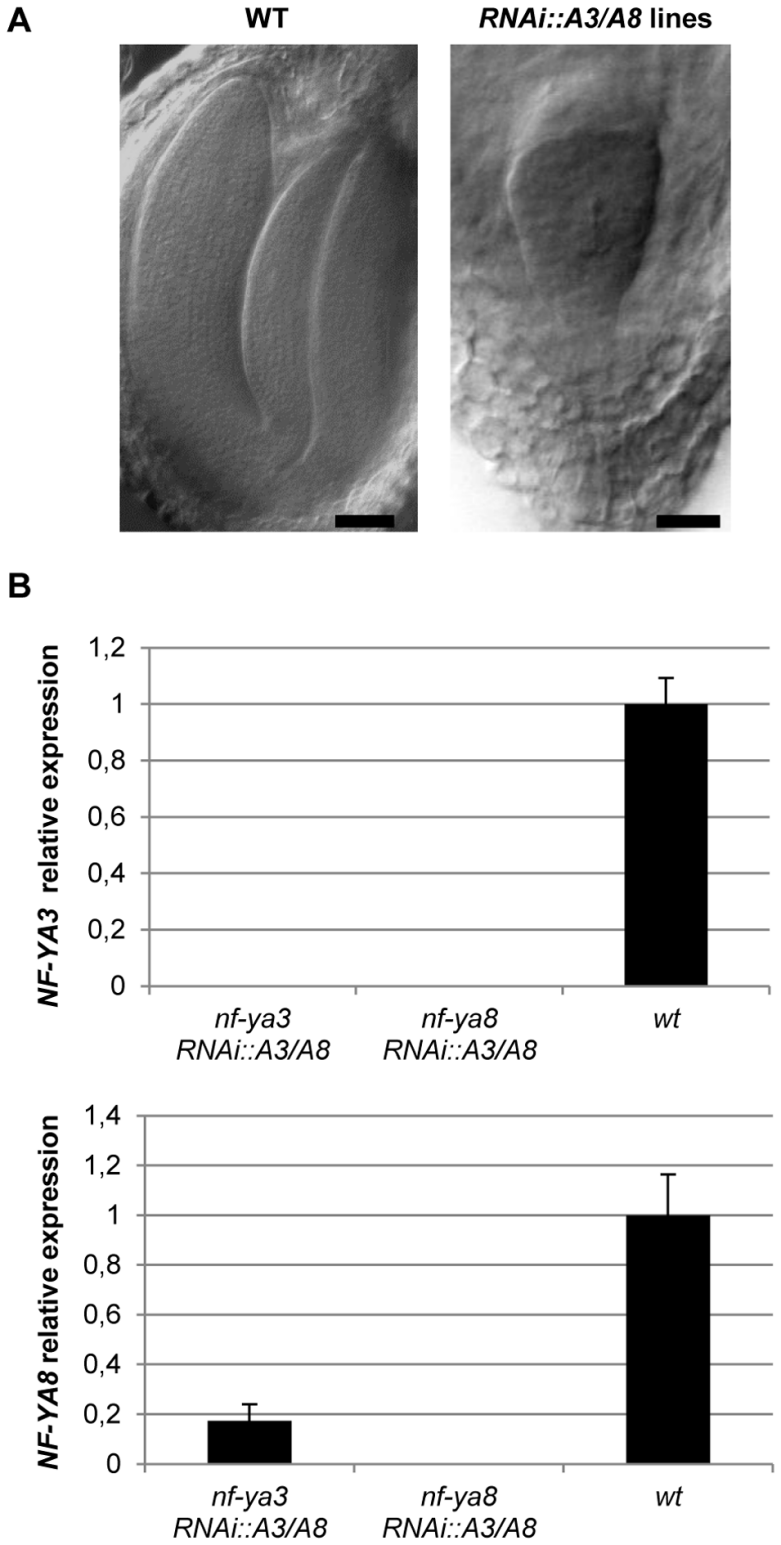
Phenotype of *RNAi::A3/A8* embryos and quantitative real-time RT-PCR analyses. (A) Cleared seeds viewed with Nomarski optics. Wild-type embryo at cotyledon stage and defective arrested embryos present in the same siliques at 10 DAP from *nf-ya3 RNAi::A3/A8* and *nf-ya8 RNAi::A3/A8* lines. Bars = 50 µm. (B) Expression levels of *NF-YA3* and *NF-YA8* genes in *RNAi::A3/A8* lines. Total RNAs were extracted from siliques of wild-type and of *nf-ya3 RNAi::A3/A8* and *nf-ya8 RNAi::A3/A8* plants and analyzed by real time RT-PCR. Wild type siliques were used as calibrator for relative expression levels for each gene analyzed. Bars represent standard deviations of measurements performed in triplicate in three biological replicates.

A post-transcriptional silencing approach mediated by RNA interference (RNAi) was carried out to verify whether the embryo lethal phenotype is due to the mutations in both *NF-YA3* and *NF-YA8* genes. A 200-bp specific fragment for *NF-YA8* that shared 80% of identity only with *NF-YA3* was used to suppress the *NF-YA3* and *NF-YA8* gene expression at the same time. Single mutants *nf-ya3* and *nf-ya8* were transformed with the RNAi construct specific for *NF-YA3* and *NF-YA8* under the control of the *35S* promoter and T_1_ primary transformants were recovered on hygromycin selection. Four out of 51 T_1_ *nf-ya3 RNAi::A3/A8* transgenic lines and four out of 49 T1 *nf-ya8 RNAi::A3/A8* transgenic lines produced defective T_2_ seeds. More specifically, T_1_ plants from *nf-ya3 RNAi::A3/A8* lines segregated 5.36%, 18.18%, 17% and 24.78% arrested T2 seeds and T1 plants from *nf-ya8 RNAi::A3/A8* lines segregated 6.9%, 17.13%, 24% and 26% arrested T2 seeds. Although the RNAi::A3/A8 construct was incompletely penetrant, these results suggest that *NF-YA3* and *NF-YA8* are required for embryo development. In particular, the analysis of 10 cleared siliques collected at 10 DAP (Days After Pollination) of *nf-ya3 RNAi::A3/A8* line (24.78% of arrested seeds) and *nf-ya8 RNAi::A3/A8* line (26% of arrested seeds) showed that in the defective seeds the embryos phenocopied the *nf-ya3 nf-ya8* double mutants: the embryos proper and the suspensors were characterized by aberrant division patterns resulting in defects in the apical and basal domains (Fig. 6 A). To confirm that abnormal embryo development resulted from silencing of both *NF-YA3* and *NF-YA8* genes, we analyzed *NF-YA3* and *NF-YA8* RNA levels in the transgenic siliques by quantitative real-time RT-PCR using specific primers for the *NF-YA3* and *NF-YA8* genes. Our results indicated that siliques of *nf-ya8 RNAi::A3/A8* lines displaying embryos with the mutant phenotype possessed undetectable levels of *NFY-A3* and *NF-YA8* and siliques of *nf-ya3 RNAi::A3/A8* line possessed a low level of *NF-YA8* and an undetectable level of *NF-YA3* (Fig. 6 B). Despite both *NF-YA3* and *NF-YA8* were essentially undetectable in seeds of these *RNAi* lines, no post-embryonic phenotypes, such as aberrant seedlings, or other phenotypic alterations in plant development were observed in T_2_ plants. Together, these results suggest that concurrent suppression and reduction of *NF-YA3* and *NF-YA8* gene expression induced defects in the embryo development.

### Partial rescue of nf-ya3 nf-ya8 mutant phenotype by complementation

To further demonstrate that the embryo-lethal phenotype is determined by the loss of activity of *NF-YA3* and *NF-YA8*, a complementation experiment was performed. Plants *nf-ya3/nf-ya3 NF-YA8/nf-ya8* were transformed with a construct carrying the coding sequence (CDS) of *NF-YA3* or with a construct with the coding sequence of *NF-YA8* driven by a 35S promoter (*35S::CDS-NF-YA3* and *35S::CDS-NF-YA8*). Primary T1 transformants were identified by kanamycin (KAN) selection, whereas the genotype and the presence of the complementation construct were determined by PCR.

We identified twenty-five *nf-ya3/nf-ya3 NF-YA8/nf-ya8* T_1_ plants that contained the complementation construct *35S::CDS-NF-YA3*. These plants were grown to maturity and siliques analysed to identify complemented lines. In particular, if only one copy of the complementation construct had inserted, fully complemented lines should show 6.25% (1/16) of arrested seeds, whereas partially complemented lines should show a percentage of arrested seeds between 6.25% and 25%. In the case of two copies of the construct, the arrested seeds of fully complemented lines should be 1.56% (1/64), whereas in partially complemented lines the percentage of arrested seeds should be between 1.56% and 25%. Non-complemented lines would show 25% of arrested seeds. We identified four lines that showed various levels of partial complementation (Table 3). Segregation analysis for kanamycin resistance indicated that three lines had two copies of the *35S::CDS-NF-YA3* construct (KAN^R^∶KAN^S^, 15∶1) and showed 7.5%, 13.71%, 17.53% of arrested seeds, respectively. One line, with one copy of the construct (KAN^R^∶KAN^S^, 3∶1), showed 14.29% of arrested seeds.

**Table 3:** Complementation of *nf-ya3/nf-ya3 nf-ya8/nf-ya8* double mutants with *35S::CDS-NF-YA3* construct.

		Progeny phenotype (observed/expected^b^)				
T_1_ line^a^	KAN^R^/KAN^S^	Normal seeds	Arrested seeds	Total	P value^c^	Observed arrested seeds (%)
11	15∶1	74/78.75	6/1.25	80/80	P<0.0001	7.5
13	15∶1	258/294.33	41/4.67	299/299	P<0.0001	13.71
16	15∶1	160/190.97	34/3.03	194/194	P<0.0001	17.53
48	3∶1	126/137.81	21/9.19	147/147	P<0.0001	14.29

a *nf-ya3/nf-ya3 NF-YA8/nf-ya8* T1 plants carrying the *35S::CDS-NF-YA3* complementation construct.

b Arrested seeds expected in case of full complementation.

c Chi-square test for 1/16 (one copy of construct inserted) and 1/64 (two copies of construct inserted) segregation hypothesis.

For all four lines, we then isolated by kanamycin selection *nf-ya3/nf-ya3 NF-YA8/nf-ya8* T_2_ plants homozygous for the complementation construct. Siliques of *nf-ya3/nf-ya3 NF-YA8/nf-ya8* T_3_ plants showed a percentage of arrested seeds lower than 25%, confirming a partial complementation. Green seedlings of these four lines (T_4_ generation) were analysed by PCR, which showed that some of them were indeed *nf-ya3 nf-ya8* double mutants. These results suggest that the introduced construct *35S::CDS-NF-YA3* can partially complement the embryo arrested phenotype.

The same complementation experiment was performed using the *35S::CDS-NF-YA8* construct to transform *nf-ya3/nf-ya3 NF-YA8/nf-ya8* plants. Sixty independent KAN-resistant T_1_ lines were isolated and four of them showed various levels of partial complementation (Table 4). Lines 16 and 21 carried one copy of the *35S::CDS-NF-YA8* construct and showed 18.38% and 18.86% of arrested seeds, respectively. Two copies were inserted in lines 34 and 52, that showed a reduction to 18.72% and 18.93% arrested seeds, respectively. Also in this case, for all four lines we then isolated by kanamycin selection *nf-ya3/nf-ya3 NF-YA8/nf-ya8* T_2_ plants homozygous for the complementation construct. The analysis of siliques from *nf-ya3/nf-ya3 NF-YA8/nf-ya8* T_3_ plants indicated that lines 16, 21 and 52 were partially complemented. Also in this case, PCR analysis confirmed that some of T_4_ plants were *nf-ya3 nf-ya8* double mutants. Interestingly, in line 34 which carried two copies of the complementation construct, siliques showed 100% normal seeds. No post-embryonic phenotypes, such as aberrant seedlings, or other phenotypic alterations in plant development were observed in complemented plants. These data indicate that the complementation construct was able to partially rescue the arrested embryos and that in only one line the *35S::CDS-NF-YA8* construct was sufficient to fully complement the embryo lethality.

**Table 4:** Complementation of *nf-ya3/nf-ya3 nf-ya8/nf-ya8* double mutants with *35S::CDS-NF-YA8* construct.

		Progeny phenotype (observed/expected^b^)				
T_1_ line^a^	KAN^R^/KAN^S^	Normal seeds	Arrested seeds	Total	P value^c^	Observed arrested seeds (%)
34	15∶1	178/215.58	41/3.42	219/219	P<0.0001	18.72
52	15∶1	167/202.78	39/3.22	206/206	P<0.0001	18.93
16	3∶1	182/209.06	41/13.94	223/223	P<0.0001	18.38
21	3∶1	185/213.75	43/14.25	228/228	P<0.0001	18.86

a *nf-ya3/nf-ya3 NF-YA8/nf-ya8* T1 plants carrying the *35S::CDS-NF-YA8* complementation construct.

b Arrested seeds expected in case of full complementation.

c Chi-square test for 1/16 (one copy of construct inserted) and 1/64 (two copies of construct inserted) segregation hypothesis.

**Figure 7:**
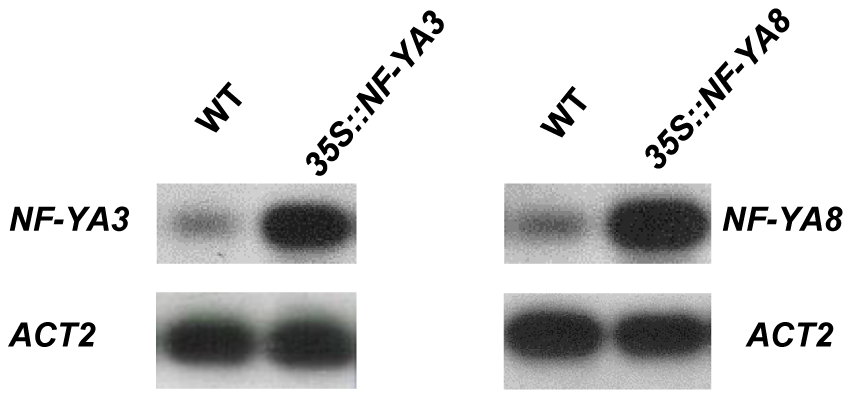
RT-PCR of *NF-YA3* and *NF-YA8* transcripts in *35S::NF-YA3* and *35S::NF-YA8*. RT-PCR analysis showing transcript levels of *NF-YA3* and *NF-YA8* in developing siliques of transgenic and wild-type plants. PCR products were blotted and hybridized with gene-specific random primed probes. The *ACTIN 2* (*ACT2*) gene was used as a control.

In parallel, transgenic plants overexpressing *NF-YA3* or *NF-YA8* were obtained by transforming the wild-type plants with the same constructs used for the complementation. RT-PCR showed that the *NF-YA3* or *NF-YA8* transcript levels in overexpression lines were higher than in wild-type (Fig. 7). Phenotypic analysis and detailed morphological analysis of these overexpression lines carried out by Nomarski microscopy did not reveal morphological defects in seedlings or plant development and in ovule, pollen and embryo development (data not shown).

### Auxin response is altered in the nf-ya3 nf-ya8 embryos

**Figure 8:**
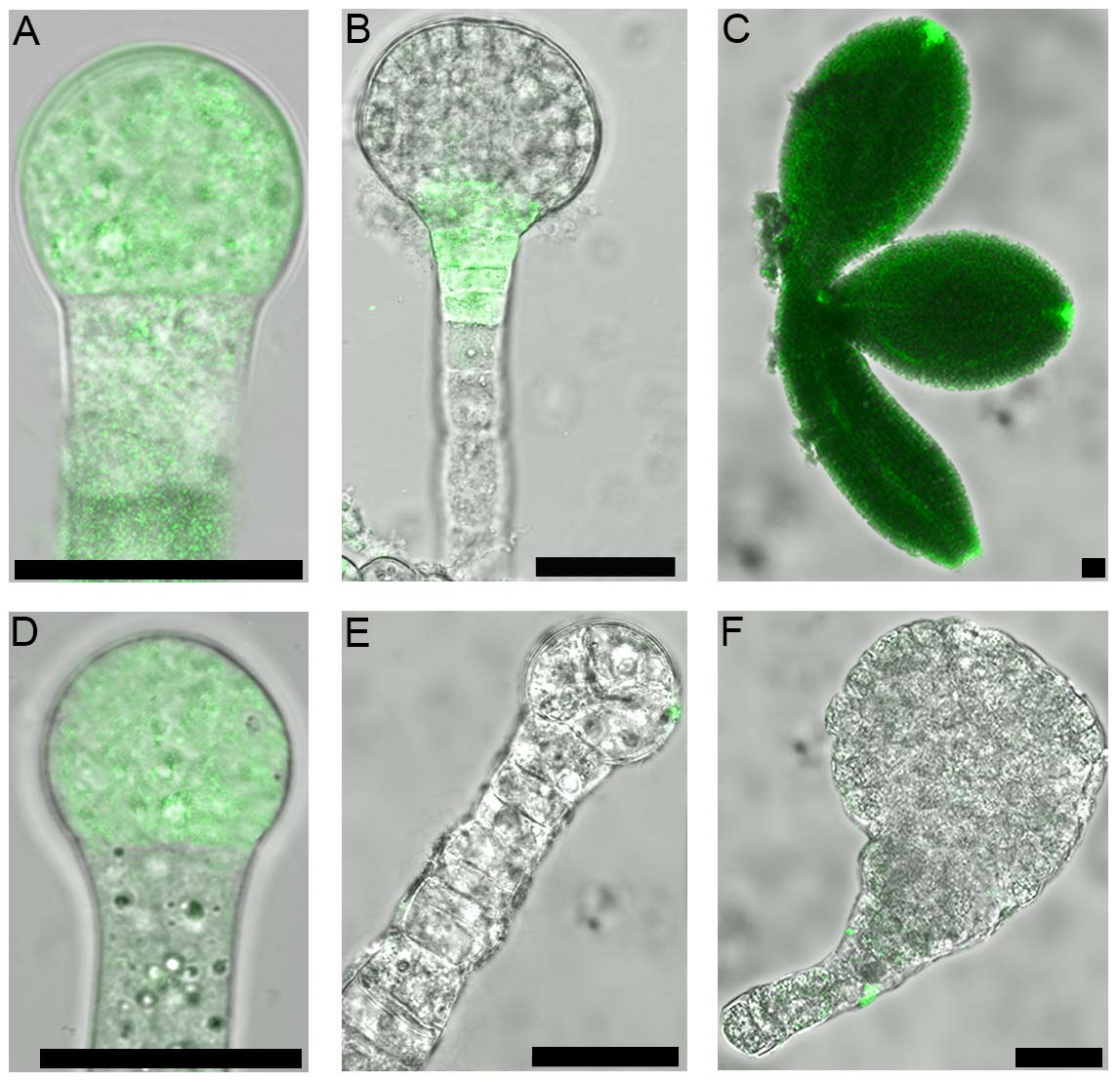
Expression pattern of DR5rev::GFP in wild-type and nf-ya3 nf-ya8 embryo. (A,B,C) *DR5rev::GFP* expression in wild-type embryos. Embryo at proembryo stage (A), globular stage (B) and at cotyledon stage (C). (D,E,F) *DR5rev::GFP* expression in *nf-ya3 nf-ya8* defective embryos. Confocal laser scanning images; bars = 25 µm.

Defective cell division plans in *nf-ya3 nf-ya8* embryos raised the question whether auxin response was affected in *nf-ya3 nf-ya8* embryos. To address this question, we crossed the auxin response marker line *DR5rev::GFP* to *nf-ya3/nf-ya3 NF-YA8/nf-ya8* plants. The *DR5* synthetic auxin-responsive promoter is used to visualize the spatial pattern of auxin response and indirectly the auxin distribution and it is specifically active from the early embryo stages [43]. Our analysis revealed that in wild-type embryos GFP signal was restricted to the developing proembryo (Fig. 8A), whereas from the 32-cell globular stage onwards it was restricted to the hypophyseal region (Fig. 8 B). No GFP signal was detected in *nf-ya3 nf-ya8* defective embryos (Fig. 8 E), whereas normal GFP signal was detected in earlier stages (Fig. 8D). Furthermore, in wild-type embryos at later stages GFP signal was highly detected in the tips of the developing cotyledons and in the provascular strands (Fig. 8 C), whereas again no GFP signal was observed in *nf-ya3 nf-ya8* embryos at later stages showing aberrant cell divisions and development (Fig. 8 F). We can conclude that defects in the *nf-ya3 nf-ya8* embryos are associated to a defective auxin response.

## Discussion

### NF-YA3 and NF-YA8 are functionally redundant regulators of embryo development

In this study, using a reverse genetic approach, we have shown that the Arabidopsis *NF-YA3* and *NF-YA8* control embryo development. The GUS expression analysis of the *pNF-YA3::GUS* and *pNF-YA8::GUS* transgenic lines revealed that both genes are expressed in vegetative and reproductive tissues. In particular, GUS staining was low during pollen and ovule development, whereas it was high during embryo development from the globular embryo stage to the torpedo stage and then decreased in the mature embryo. *In situ* hybridization essentially confirmed these GUS expression results, except for endosperm, where a clear GUS signal was missing. Since GUS results were obtained by cloning putative *NF-YA3* and *NF-YA8* promoter regions upstream of the *GUS* reporter gene, they may not exactly reflect the real expression pattern of genes, because lacking endosperm-specific regulatory elements possibly located in 3′ untranslated regions (3′UTR) or introns not included in the constructs. Interestingly, previous GUS expression analyses from Siefers and colleagues [1]of *NF-YA3* and *NF-YA8* revealed that GUS staining was undetectable in flowers, in root tips and low in rosette leaves. We suggest that these differences in GUS expression results are due to differences in the promoter::GUS fusions used. For *NF-YA3*, the promoter region used by Siefers and colleagues [1] was 995 bp from ATG, whereas we used a longer putative promoter (1995 bp from ATG) corresponding to the entire upstream intergenic region. For *NF-YA8*, both promoters included the entire intergenic region including the 3′UTR of the upstream gene, but there was a subtle difference in length (995 bp vs 1000 bp), that may be relevant. Since our *in situ* analyses essentially confirmed the GUS expression results presented here, we concluded that the genomic region upstream of the start codon fused to the *GUS* reporter gene contained all the regulatory sequences essential for the gene expression of *NF-YA3* and *NF-YA8*, except those for endosperm expression, and that the genes may have an hypothetical function during embryo development.

Identification of the factors that control embryogenesis is of significant interest, since they are essential to produce a viable plant with a normal phenotype under diverse conditions. Therefore, several large-scale screenings have been performed to identify these factors [59]. Embryo mutants are typically identified using two criteria: reduced seed set and segregation distortion [60]. The *nf-ya3* and *nf-ya8* single mutants did not show any phenotypic defect and the segregation analyses revealed that the observed genotypic ratios were not statistically different from the expected. However, in the siliques produced by *nf-ya3/nf-ya3 NF-YA8/nf-ya8* plants we observed a number of arrested seeds higher than in wild-type plants and the segregation of *nf-ya3/nf-ya3 NF-YA8/NF-YA8* versus *nf-ya3/nf-ya3 NF-YA8/nf-ya8* genotypic classes was 1:2, whereas the double mutants were totally absent. Detailed analysis of siliques produced by *nf-ya3/nf-ya3 NF-YA8/nf-ya8* plants showed that 25% of the seeds were arrested and this percentage correlated with the double mutant genotypic class. The fact that neither single mutant displayed a phenotype, while the *nf-ya3 nf-ya8* double mutant was embryo lethal, implies that *NF-YA3* and *NF-YA8* are functionally redundant, which is not surprising considering the high homology they share and that these genes lie within segmental chromosomal duplications. Taken together, these results indicate that *NF-YA3* and *NF-YA8* have an essential function during embryogenesis.

### NF-YA3 and NF-YA8 are required for early embryogenesis

The morphological analysis performed by Nomarski microscopy on siliques produced by *nf-ya3/nf-ya3 NF-YA8/nf-ya8* plants revealed the specific role of *NF-YA3* and *NF-YA8* during embryo development. In particular all the arrested seeds analysed contained abnormal embryos, in which the division plan of cells and number of cells in proembryo and suspensor were incorrect. Therefore, the mutant embryo showed an abnormal division of the uppermost region of the suspensor close to the proembryo (i.e. the hypophysis cell). These morphological defects suggests that *NF-YA3* and *NF-YA8* have a role during early embryogenesis.

In plants, axis formation involves the signaling molecule auxin, a plant hormone that has a gradient of accumulation and distribution during the time of development and that is responsible for structural plant organization and for correct plan determination [61]. PINFORMED-dependent asymmetric auxin efflux and the maintenance of normal auxin gradients within the embryo are necessary for the correct apical-basal axis determination in the embryo [39]. PIN1 and PIN7 localization correlated with the apical-basal auxin gradients during early embryogenesis. After zygotic division, auxin flux is initially directed up into the apical cell by PIN, that localizes to the apical end of the basal daughter cell; moreover, PIN1 marks the apical cell and the proembryo cell boundaries in a non-polar fashion. At the 32-cell stage, auxin undergoes apical-to-basal transport; it is transported into the future hypophysis and this event is marked by the shift of PIN1 localization in the basal membranes of the provascular cells facing the hypophysis and by the reversal of PIN7 polarity in the basal ends of suspensor cells. Mild defects at the basal embryo pole were observed in the *pin1* mutant. In *pin7* mutants the embryos fail to establish the apical-basal auxin gradient and the specification of the apical daughter cell of the zygote is compromised because there is a horizontal instead of vertical division. In most cases, the defects are confined to the lower region of the proembryo and in some cases two proembryos developed on top of each other: the lower maintained the morphology of the suspensor and the limit between apical and basal structures was not defined. At the globular stage *pin7* mutant shows a recovery of normal phenotype; then, the defects are confined to the basal part of the embryo. Other *PIN* genes are expressed during embryogenesis; *PIN3* in the basal pole of the heart stage embryo and *PIN4* in the basal pole of the globular stage embryo after reversal of the gradient, that is in the progeny cells of the hypophysis and in the provascular initial cells of the root meristem. These genes are redundant and the recovery of *pin7* corresponds with PIN1 basal localization, *PIN4* expression and reversal of auxin gradient. Interestingly, *pin1 pin3 pin4 pin7* mutants failed to be recovered and showed serious defects in apical-basal embryo axis determination producing aberrant filamentous globular embryos [43].

Promoter-reporter gene studies using *DR5rev::GFP* revealed that the GFP signal was completely absent in *nf-ya3 nf-ya8* defective embryos, except at proembryo stage when they displayed a normal embryo phenotype. These findings suggest that mutations in *NF-YA3* and *NF-YA8* may interfere with auxin response in the embryos. In particular, a first possibility is that the inhibition and/or alteration of specific cell divisions in the proembryo and in the suspensor may affect the auxin transport by PIN carriers, leading to embryo patterning defects. In order to validate this hypothesis it will be necessary to check the correct localization of PIN proteins in the mutant embryos. A second possibility is that the *NF-Y* and *PIN* genes could have a relationship during early stages of embryo development. The NF-YA3 and NF-YA8 redundant subunits may form equivalent NF-Y complexes with NF-YB and NF-YC subunits [17], [62], which could directly or indirectly regulate the expression of *PIN* genes, thus controlling the regulation of directional auxin transport implicated in embryo developmental processes. Further studies will be necessary to understand whether *NF-Y* and *PIN* genes may act together in the determination of the auxin distribution in developing embryos.

### Complementation and RNAi experiments showed that NF-YA3 and NF-YA8 are required from the globular stage of embryo development

The complementation experiment performed on *nf-ya3/nf-ya3 NF-YA8/nf-ya8* plants revealed that the *35S::CDS-NF-YA3* construct can partially rescue the arrested phenotype of *nf-ya3 nf-ya8* mutant embryos. Similarly, when we used the *35S::CDS-NF-YA8* construct to complement *nf-ya3 nf-ya8* double mutants, we found three partially complemented lines and one line fully complemented, showing siliques with totally normal seeds. The partial complementation may be due to a low efficient expression of the *CaMV 35S* promoter during the early stages of embryo development. The *35S* promoter has been previously used to complement embryo lethal mutants in Arabidopsis [63]–[65]. However, it was reported that the *CaMV 35S* promoter is not active in globular stage embryos, but it is activated in the heart stage embryo [62], [66], [67]. Possibly, a better complementation experiment would be achieved by using the endogenous *NF-YA3* and *NF-YA8* putative promoters or the *RPS5A* promoter [68]. Nonetheless, in this work we showed that overexpressing NF-YA3/NF-*YA8* under the *35S* promoter was sufficient to partially rescue *nf-ya3 nf-ya8* abnormal globular embryos, thus suggesting that NF-YA3 and NF-YA8 are required from the globular stage of embryo development, since abnormal cell division plans can be already observed at the early globular stage. This hypothesis is supported by the GUS expression experiment and the *in situ* analyses, showing that *NF-YA3* and *NF-YA8* are already expressed at low levels in the early-globular stage and at higher levels in the globular-heart stage of embryo development.

Interestingly, the suppression of *NF-YA3* and *NF-YA8* gene expression by RNAi experiments confirmed the complementation results. The RNAi construct showed incomplete penetrance probably because, also in this case, it is controlled by the *35S* promoter, the same used for complementation analyses. The small fraction of RNAi transgenic lines showing embryo defects likely reflects the weak activity of *35S* promoter in globular embryo stages. However, although not all embryos of *RNAi::A3/A8* transgenic plants were arrested, we showed that the suppression of the two *NF-YA* genes produced defective embryos with mutant phenotypes similar to those of T-DNA insertional double mutant embryos. Moreover, results obtained from quantitative real-time RT-PCR are in agreement with this conclusion. Analysis of *RNAi::A3/A8* lines showed that, in transgenic siliques, the absence or high reduction of *NF-YA3* and *NF-YA8* endogenous transcripts, respectively, are necessary and sufficient to obtain abnormal globular embryos.

## Conclusions

Our results show that *NF-YA3* and *NF-YA8* are functionally redundant genes required for Arabidopsis early embryogenesis. In particular, we have shown that the *nf-ya3 nf-ya8* embryos have an irregular globular shape showing defects in cell divisions in both embryo proper and suspensor. We also found that mutations in both *NF-YA3* and *NF-YA8* affect the auxin response in early developing embryo, known to be involved in embryo axis establishment by its polar transport. Finally, complementation and RNA interference experiments demonstrated that *NF-YA3* and *NF-YA8* are new functionally redundant genes specifically required from the globular stage of embryo development.

## Supporting Information

Figure S1 **In situ analyses using NF-YA3 and NF-YA8 sense probes in developing flower and seed.** No hybridization signal was detected in developing flowers (A, C) and embryo at 10 DAP (B, D) using *NF-YA3* (A, B) and *NF-YA8* (C, D) sense probes. Bars = 50 µm.
(TIF)
