# Supplementary figures and images for: The Arabidopsis NF-YA3 and NF-YA8 Genes Are Functionally Redundant and Are Required in Early Embryogenesis
Source: PLoS One. 2013 Nov 26;8(11):e82043. doi: 10.1371/journal.pone.0082043 (PMC3841131; doi:10.1371/journal.pone.0082043)

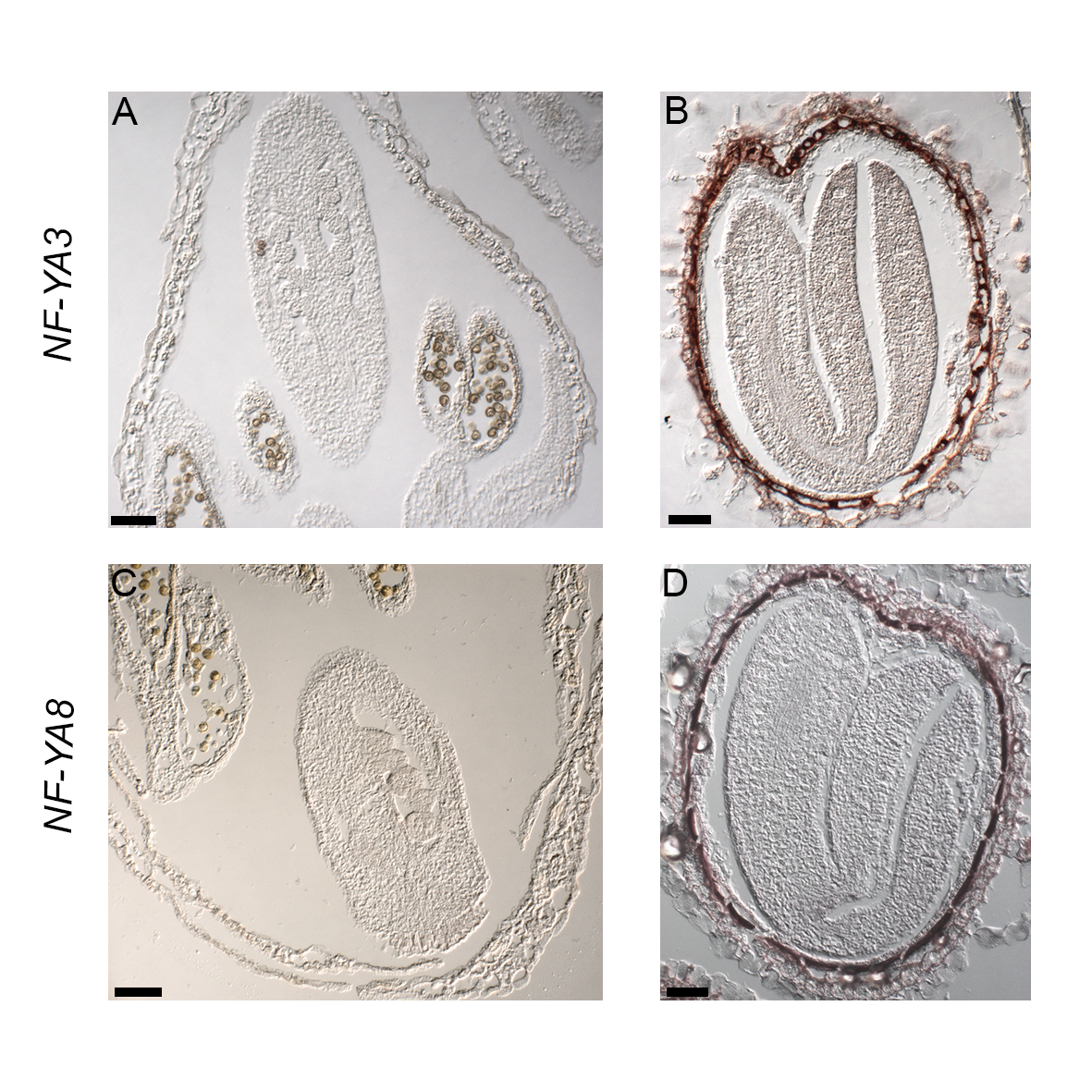

Supplement: Figure S1 — In situ analyses using NF-YA3 and NF-YA8 sense probes in developing flower and seed. No hybridization signal was detected in developing flowers (A, C) and embryo at 10 DAP (B, D) using NF-YA3 (A, B) and NF-YA8 (C, D) sense probes. Bars = 50 µm. (TIF) [file pone.0082043.s001.tif]
